# Identification of key predictors of hospital mortality in critically ill patients with embolic stroke using machine learning

**DOI:** 10.1042/BSR20220995

**Published:** 2022-09-14

**Authors:** Wei Liu, Wei Ma, Na Bai, Chunyan Li, Kuangpin Liu, Jinwei Yang, Sijia Zhang, Kewei Zhu, Qiang Zhou, Hua Liu, Jianhui Guo, Liyan Li

**Affiliations:** 1 Institute of Neuroscience, Kunming Medical University, Kunming, Yunnan, China; 2 Department of Neurology, Nanbu People’s Hospital, Nanbu, Sichuan, China; 3 Department of Neurology, The Third People’s Hospital of Chengdu and The Affiliated Hospital of Southwest Jiaotong University, Chengdu, Sichuan, China; 4 Department of Neurology, The Second Affiliated Hospital of Kunming Medical University, Kunming, Yunnan, China; 5 Second Department of General Surgery, First People’s Hospital of Yunnan Province, Kunming, Yunnan, China

**Keywords:** feature selection, machine learning, MIMIC-IV, prediction model, random forest

## Abstract

Embolic stroke (ES) is characterized by high morbidity and mortality. Its mortality predictors remain unclear. The present study aimed to use machine learning (ML) to identify the key predictors of mortality for ES patients in the intensive care unit (ICU). Data were extracted from two large ICU databases: Medical Information Mart for Intensive Care (MIMIC)-IV for training and internal validation, and eICU Collaborative Research Database (eICU-CRD) for external validation. We developed predictive models of ES mortality based on 15 ML algorithms. We relied on the synthetic minority oversampling technique (SMOTE) to address class imbalance. Our main performance metric was area under the receiver operating characteristic (AUROC). We adopted recursive feature elimination (RFE) for feature selection. We assessed model performance using three disease-severity scoring systems as benchmarks. Of the 1566 and 207 ES patients enrolled in the two databases, there were 173 (15.70%), 73 (15.57%), and 36 (17.39%) hospital mortality in the training, internal validation, and external validation cohort, respectively. The random forest (RF) model had the largest AUROC (0.806) in the internal validation phase and was chosen as the best model. The AUROC of the RF compact (RF-COM) model containing the top six features identified by RFE was 0.795. In the external validation phase, the AUROC of the RF model was 0.838, and the RF-COM model was 0.830, outperforming other models. Our findings suggest that the RF model was the best model and the top six predictors of ES hospital mortality were Glasgow Coma Scale, white blood cell, blood urea nitrogen, bicarbonate, age, and mechanical ventilation.

## Introduction

According to the systematic analysis for the Global Burden of Disease Study 2019, stroke was the second-leading cause of death and the third-leading cause of disability and death [1]. Ischemic stroke (IS) accounts for ∼62.4% of all new-onset strokes [1]. Embolic stroke (ES), also called cerebral embolism, is the most common subtype of IS and the most rapidly developing type of all strokes [2,3]. Furthermore, compared with other stroke subtypes, ES has a higher disease severity, a poorer prognosis, and a higher recurrence rate [4]. Therefore, ES is a huge burden on society due to reduced quality of life, lost productivity, premature mortality, and intangible costs, particularly for the critically ill patients in the intensive care unit (ICU) [5,6].

Prediction and prognosis are central to medicine. All diagnostic and therapeutic measures aim at improving prognosis outcomes [7]. Clinicians need to make predictions on the disease prognosis to support their decision-making. However, it is challenging for clinicians to accurately assess the disease severity and predict the outcome in patients with various clinical data based on initial intuition. Consequently, several disease-severity scoring systems have been developed over the last few decades, such as the Acute Physiology and Chronic Health Evaluation III (APACHE III) [8], Sequential Organ Failure Assessment (SOFA) score [9], and Oxford Acute Severity of Illness Score (OASIS) [10]. The scoring systems are intrinsically prediction models. They combine a variety of variables that represent predictors of disease severity to predict the prognostic outcome. Due to their predictive validity, they gradually became essential tools for clinical decision-making.

A notable fact is that almost all scoring systems depend on linear models to identify relevant predictors. With the increase in clinical data on potential predictors of outcome, nonlinearity may exist. Scoring systems may fail to capture nonlinear relationships and complex interactions among predictors. Moreover, scoring systems artificially discretize continuous variables, which may cause the loss of predictive information [11]. Additionally, they are generalistic severity of illness scoring systems that are not targeted to specific diseases; therefore, they have shown mixed prediction accuracy in different diseases [12], high for some diseases, such as sepsis, but low for most diseases, such as craniocerebral diseases [13–15].

In recent years, machine learning (ML) applications have grown in popularity across a wide range of domains [16]. ML makes fewer assumptions in prediction modeling than traditional statistical approaches. It is a demonstrably powerful technique because it can handle a high number of features (i.e., predictors), consider all possible permutations, and learn nonlinear relationships and interactions without requiring a predetermined linear relationship input by the researcher [17,18]. Feature selection is an important aspect in practical applications of ML. It reduces the dimensionality by choosing relevant features and eliminating irrelevant features to improve the prediction performance of the predictors and provide cost-effective predictors [19].

ML has been successfully used to aid clinical diagnosis and improve the ability to predict patient outcomes [20], while the feature selection strategies have often been used to identify key prognosis risk factors, genes, and proteins for diseases [21,22]. However, as far as we know at present, there has been no ML-based study to explore the mortality predictors for ES patients to date. Therefore, the present study attempts to apply multiple ML algorithms to the data derived from the ES patient cohorts of two large ICU databases. Through rigorous ML modeling and feature selection techniques, we hope to build an ML model that has a better prediction performance for critically ill ES patients than the commonly used scoring systems and identify the key mortality predictors.

## Materials and methods

### Data sources

This is a retrospective cohort study with the data of ES patients derived from two public ICU databases: Medical Information Mart for Intensive Care (MIMIC)-IV [23] and eICU Collaborative Research Database (eICU-CRD) [24]. MIMIC-IV is an updated version of the MIMIC-III database and contains comprehensive clinical data of patients admitted to the ICU at Beth Israel Deaconess Medical Center between 2008 and 2019. eICU-CRD is a multicenter database with de-identified health data for >200,000 admissions to ICU in the United States between 2014 and 2015. An author (Wei Liu) of the present study was granted access to the databases (Record ID: 36180968). The present study is reported according to the guidelines of the Transparent Reporting of a multivariable prediction model for Individual Prognosis Or Diagnosis (TRIPOD) statement [25]. Informed consent is not required because all the health information data are anonymous.

### Selection of participants

To describe and analyze the two databases, we used PostgreSQL (version 13) and Navicat Premium (version 15). An author (Wei Liu) extracted the data, which was then double-checked by another author (Wei Ma) of the present study. The search terms are presented in Supplementary Material Table S1. Patients’ exclusion criteria were as follows: (i) age <18 years, (ii) not first ICU admission, (iii) inability to obtain Acute Physiology Score III (APSIII), OASIS, SOFA, and Glasgow Coma Scale (GCS) scores and (iv) missing outcome (death or survival).

### Predictors and outcome

Most disease-severity scoring systems, such as APSIII, SOFA, and OASIS, collect data from the first 24 h after admission. We thus extracted predictors during the patient’s first 24 h in the ICU. We initially included 90 readily available candidate predictors based on literature reviews [26,27], expert clinical opinion, and clinical availability in practice. After removing predictors with a missing proportion of >20%, we included a total of 58 predictors in both databases (see Supplementary Material Table S2). In our analysis, we used the mean values for some predictors that were measured multiple times to determine the central tendency of the patients’ condition. The outcome was hospital mortality, defined as the vital status for survival or death at hospital discharge.

### Model building and tuning

ML methods were performed using R software (version 4.1.3). The ‘caret’ package (version 6.0-92) was used for model building, tuning, and validation [28]. We build 15 ML models to improve the probability of identifying the best ML model, including linear discriminant analysis (LDA), quadratic discriminant analysis (QDA), partial least squares discriminant analysis (PLSDA), logistic regression (LR), least absolute shrinkage and selection operator (LASSO), naive Bayes (NB), and support vector machine (SVM) with three types of kernel: a radial basis function kernel (SVM-R), a linear kernel (SVM-L), a polynomial kernel (SVM-P), k-nearest neighbor (KNN), C5.0 decision tree, repeated incremental pruning to produce error reduction (RIPPER), random forest (RF), extreme gradient boosting machine (XGBoost), and neural network (NNET). For models with tuning parameters that needed optimization, we adjusted parameters via random search with tune length of 15 whenever applicable.

### Data preprocessing

As most ML models cannot handle missing data [29], we performed a multiple imputation model using multiple imputation by chained equation (MICE) in the R software [30]. Dummy variables were created for categorical predictors. Some models benefit from the predictors that are on a common scale and with reduced skewness (LDA, QDA, PLSDA, KNN, LR, LASSO, NB, SVM, and NNET); therefore, data were scaled, centered, and BoxCox transformed [29]. No data transformation was performed for the tree-based models (C5.0, RF, and XGBoost) and rule-based models (RIPPER).

### Model improvement and evaluation

Data distribution from MIMIC-IV and eICU-CRD is unbalanced: the survival and death groups ratio are about 1:5. The imbalance may result in a classification bias toward the majority class [31]. Therefore, we used the synthetic minority oversampling technique (SMOTE) to tackle the imbalance [32]. All models were subjected to three repetitions of 10-fold cross-validation to evaluate their performance. Receiver operating characteristic (ROC) curves were plotted, and the area under the ROC (AUROC) was used as the performance metric because of the data imbalance [33]. The best threshold of the AUROC (BTOA), area under the precision-recall curve (AUPRC), accuracy, positive predictive value (PPV), negative predictive value (NPV), F1-score, and Cohen’s kappa value were also reported. LR was used to construct models of the scoring systems as benchmarks for evaluating ML models.

### Feature selection

Feature selection was performed using recursive feature elimination (RFE) algorithm with the function ‘rfe’ in R package ‘caret’ (version 6.0-92) for the 14 ML models (LASSO was excluded because of its inherent feature selection function) [34]. RFE begins by building a model on the entire set of features, and the importance of each feature is calculated either using the provided ML model (e.g., some algorithms like RF offer importance scores) or by using a statistical method (e.g., the AUROC). Then, the least important features are removed, the model is re-built, and importance scores are computed again. This procedure is recursively repeated until the desired number of feature subsets is eventually reached. However, considering the highly time-consuming calculation of RFE, the feature subset sizes we ran contained ten items: 2, 3, 4, 5, 6, 10, 20, 35, 45, and 58.

### Model interpretation

The feature selection models can be regarded as the global interpretability method and can help understand the predictors and their overall relationship with the outcome; however, they cannot realize individual predictions. Therefore, we used the local interpretable model-agnostic explanation (LIME) to explain the impact of key features at the individual level [35]. LIME explains the classifier using a local linear model to approximate key features. Its output is an explanation list, indicating the contribution of key features to the outcome in an individual patient.

### Statistical analysis

Baseline characteristics were compared between the survival and death groups to determine their baseline comparability. The Kolmogorov–Smirnov test was used to determine normality. Continuous variables were expressed as the mean [standard deviation (SD)] (normal) or the median [interquartile range (IQR)] (non-normal). Categorical variables were expressed as the total number (percentages). The Student’s *t*-test (normal) or rank-sum test for continuous variables (non-normal) and chi-square test for categorical variables were used. All statistical analyses were performed in R (version 4.1.3). A two-tailed *P*-value < 0.05 was considered statistically significant.

## Results

### Baseline characteristics

**Figure 1:**
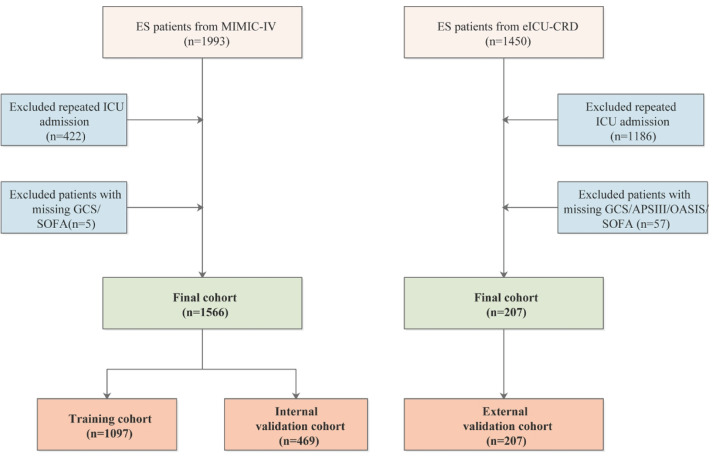
Flow chart of participants selection

Figure 1 showed the flow chart of participants’ selection in the present study. A total of 1566 and 207 ES patients were enrolled in the MIMIC-IV and eICU-CRD cohorts, respectively. The MIMIC-IV cohort was randomly split into a training cohort (70%, *n*=1097) and an internal validation cohort (30%, *n*=469). The eICU-CRD cohort was used as an external validation cohort. Demographics and baseline characteristics between the survival and death groups are summarized in Table 1. In the present study, there were 173 (15.70%), 73(15.57%), and 36 (17.39%) hospital mortality in the training cohort, internal validation cohort, and external validation cohort, respectively. There were 37 variables with statistically significant differences between the survival and death groups at the MIMIC-IV baseline, 15 variables at the eICU-CRD baseline, only 13 variables in both the databases, including APSIII, SOFA, OASIS, GCS, heart rate, red cell volume distribution width (RDW), blood urea nitrogen (BUN), creatinine, potassium, endocarditis, thrombocytopenia, coagulopathy, and mechanical ventilation (MV).

**Table 1:** Baseline characteristics of the MIMIC-IV and eICU-CRD cohorts

Patient characteristics	MIMIC IV cohort (*n*=1566)						eICU-CRD cohort (*n*=207)		
	Training set (*n*=1097)			Internal validation set (*n*=469)			External validation set (*n*=207)		
	Death, *n*=173	Survival, *n*=924	*P*	Death, *n*=73	Survival, *n*=396	*P*	Death, *n*=36	Survival, *n*=171	*P*
**Demographic characteristics**									
Age (years)	80.00	71.00	<0.001	77.00	72.00	0.016	69.50	72.00	0.972
	[67.00, 86.00]	[61.00, 82.00]		[67.00, 87.00]	[60.00, 83.25]		[56.25, 83.00]	[59.00, 80.00]	
Weight (kg)	74.20	76.10	0.120	75.00	77.25	0.618	75.67	79.65	0.193
	[62.30, 88.30]	[63.58, 91.05]		[63.90, 88.25]	[64.97, 90.05]		[62.75, 85.12]	[69.40, 92.78]	
Gender (*n*, %)			0.424			∼1			
Women	96 (55.5)	479 (51.8)		36 (49.3)	197 (49.7)		20 (55.6)	87 (51.2)	0.769
Man	77 (44.5)	445 (48.2)		37 (50.7)	199 (50.3)		16 (44.4)	83 (48.8)	
Ethnicity (%)			0.017			0.060			
Caucasian	102 (59.0)	584 (63.2)		40 (54.8)	257 (64.9)		31 (86.1)	139 (81.3)	0.691
African American	10 (5.8)	94 (10.2)		6 (8.2)	43 (10.9)		1 (2.8)	14 (8.2)	
Asian	6 (3.5)	20 (2.2)		2 (2.7)	12 (3.0)		0 (0.0)	3 (1.8)	
Hispanic/Latino	3 (1.7)	35 (3.8)		2 (2.7)	18 (4.5)		2 (5.6)	6 (3.5)	
Others/Unknown	52 (30.1)	191 (20.7)		23 (31.5)	66 (16.7)		2 (5.6)	9 (5.3)	
**Vital signs**									
SBP (mmHg)	123.50	130.00	0.001	129.00	130.00	0.560	126.33	131.74	0.267
	[109.00, 142.00]	[117.00, 144.00]		[115.00, 146.00]	[118.00, 144.75]		[111.49, 141.40]	[118.51, 147.52]	
DBP (mmHg)	64.00	70.00	<0.001	64.00	68.00	0.004	69.58	69.96	0.868
	[56.75, 74.25]	[60.00, 78.00]		[57.00, 74.00]	[61.00, 80.00]		(11.24)	(12.22)	
Heart_Rate (beat/min)	88.50	80.00	<0.001	87.00	80.00	0.003	92.97	81.24	0.002
	[74.75, 100.00]	[70.00, 91.00]		[74.00, 97.00]	[70.00, 91.00]		[81.31, 102.44]	[72.94, 93.00]	
Respiratory_Rate (breaths/min)	21.00	19.00	<0.001	19.00	19.00	0.119	19.74	18.18	0.092
	[18.00, 23.00]	[17.00, 21.00]		[18.00, 22.00]	[17.00, 21.00]		[17.71, 21.71]	[16.39, 21.00]	
Temperature (°C)	36.90	36.80	0.338	36.90	36.90	0.175	37.17	36.80	0.010
	[36.60, 37.30]	[36.70, 37.10]		[36.70, 37.40]	[36.70, 37.10]		[36.79, 37.40]	[36.63, 37.14]	
SpO_2_ (%)	97.10	96.90	0.254	97.70	97.00	0.018	97.65	97.25	0.215
	[95.65, 98.70]	[95.80, 98.10]		[96.30, 99.30]	[96.00, 98.30]		[96.48, 99.18]	[95.59, 98.43]	
**Laboratory tests**									
WBC(10^9^/L)	12.25	9.90	<0.001	12.25	9.80	<0.001	3.94	4.10	0.227
	[9.03, 15.79]	[8.00, 12.90]		[9.61, 16.85]	[7.70, 12.75]		(0.64)	(0.69)	
RBC(10^12^/L)	3.66	4.04	<0.001	3.76	3.93	0.035	3.89	4.12	0.205
	[3.13, 4.30]	[3.47, 4.51]		[3.09, 4.27]	[3.44, 4.42]		[3.45, 4.33]	[3.64, 4.60]	
MCH (pg)	30.18	30.00	0.402	30.12	30.05	0.726	30.08	30.02	0.713
	[28.55, 31.50]	[28.60, 31.40]		[28.70, 31.45]	[28.60, 31.35]		[28.81, 31.88]	[28.71, 31.50]	
MCHC (g/dL)	32.64	33.00	<0.001	32.76	32.97	0.157	32.87	33.07	0.376
	[31.51, 33.50]	[32.03, 34.00]		[31.55, 33.81]	[32.10, 33.80]		(1.25)	(1.20)	
RDW (%)	14.72	13.97	<0.001	14.42	14.07	0.046	15.00	14.15	0.023
	[13.66, 16.54]	[13.20, 15.00]		[13.65, 15.68]	[13.20, 15.37]		[13.95, 16.03]	[13.40, 15.43]	
Hematocrit (%)	34.50	36.42	0.001	32.73	35.60	0.015	35.72	36.83	0.334
	[28.61, 38.51]	[31.20, 40.40]		[28.02, 38.01]	[30.75, 39.60]		(5.82)	(6.18)	
Hemoglobin (g/dL)	11.20	11.95	<0.001	10.59	11.70	0.009	11.78	12.18	0.319
	[9.20, 12.74]	[10.20, 13.52]		[9.07, 12.53]	[10.17, 13.25]		(2.11)	(2.16)	
Platelets (10^9^/L)	198.50	207.67	0.389	195.50	201.50	0.234	195.50	203.00	0.055
	[147.50, 268.00]	[160.00, 260.25]		[137.50, 252.50]	[162.00, 258.50]		[133.58, 237.33]	[168.12, 256.00]	
Aniongap (mEq/L)	16.00	14.50	<0.001	15.00	14.67	0.040	9.37	9.00	0.947
	[13.67, 19.00]	[12.62, 16.50]		[13.00, 18.00]	[13.00, 17.00]		[7.00, 11.50]	[7.25, 12.00]	
Bicarbonate (mEq/L)	21.80	23.50	<0.001	22.50	23.00	0.011	25.50	25.00	0.876
	[19.00, 24.50]	[21.33, 25.50]		[19.84, 23.88]	[21.00, 25.00]		[22.15, 27.25]	[23.00, 26.63]	
BUN (mg/dL)	26.84	18.33	<0.001	22.60	18.00	0.042	25.86	17.00	<0.001
	[18.46, 41.00]	[13.00, 26.00]		[14.16, 33.66]	[13.50, 26.50]		[20.50, 38.50]	[12.00, 23.50]	
Creatinine (mg/dL)	1.19	0.95	<0.001	1.10	0.97	0.167	1.16	0.90	0.001
	[0.85, 1.75]	[0.75, 1.25]		[0.78, 1.51]	[0.78, 1.35]		[0.91, 1.51]	[0.70, 1.17]	
Glucose (mEq/L)	148.00	119.00	<0.001	138.33	122.75	0.012	145.00	124.43	0.090
	[119.00, 194.92]	[102.16, 149.83]		[116.25, 172.88]	[105.38, 155.12]		[106.92, 194.90]	[106.00, 152.31]	
Chloride (mEq/L)	104.00	104.00	0.873	104.60	104.00	0.546	105.10	105.16	0.942
	[99.50, 108.00]	[101.00, 107.00]		[101.00, 107.50]	[100.50, 107.00]		(5.21)	(4.10)	
Sodium (mEq/L)	139.50	139.33	0.922	138.67	139.00	0.482	139.20	138.86	0.712
	[136.10, 142.00]	[137.00, 141.50]		[135.88, 141.22]	[136.50, 142.00]		[136.25, 141.50]	[137.00, 141.00]	
Potassium (mEq/L)	4.25	4.10	0.002	4.17	4.10	0.378	4.30	3.94	0.001
	[3.90, 4.72]	[3.80, 4.40]		[3.84, 4.48]	[3.80, 4.45]		[3.72, 4.61]	[3.67, 4.15]	
Calcium (mg/dL)	8.40	8.70	<0.001	8.43	8.70	0.001	8.41	8.59	0.145
	[7.98, 8.95]	[8.25, 9.10]		[8.05, 8.72]	[8.20, 9.05]		(0.69)	(0.66)	
**Comorbidities (%)**									
COPD			0.045			0.929			0.195
No (*n*, %)	129 (74.6)	753 (81.5)		59 (80.8)	325 (82.1)		32 (88.9)	164 (95.9)	
Yes (*n*, %)	44 (25.4)	171 (18.5)		14 (19.2)	71 (17.9)		4 (11.1)	7 (4.1)	
CHD			0.010			0.444			0.448
No (*n*, %)	113 (65.3)	694 (75.1)		57 (78.1)	289 (73.0)		30 (83.3)	153 (89.5)	
Yes (*n*, %)	60 (34.7)	230 (24.9)		16 (21.9)	107 (27.0)		6 (16.7)	18 (10.5)	
CHF			<0.001			0.146			0.673
No (*n*, %)	100 (57.8)	668 (72.3)		44 (60.3)	276 (69.7)		28 (77.8)	141 (82.5)	
Yes (*n*, %)	73 (42.2)	256 (27.7)		29 (39.7)	120 (30.3)		8 (22.2)	30 (17.5)	
Hypertension			0.653			∼1			∼1
No (*n*, %)	133 (54.1)	637 (48.3)		35 (47.9)	192 (48.5)		20 (55.6)	93 (54.4)	
Yes (*n*, %)	113 (45.9)	683 (51.7)		38 (52.1)	204 (51.5)		16 (44.4)	78 (45.6)	
Hyperlipidemia			0.049			0.003			0.809
No (*n*, %)	98 (56.6)	445 (48.2)		51 (69.9)	198 (50.0)		30 (83.3)	148 (86.5)	
Yes (*n*, %)	75 (43.4)	479 (51.8)		22 (30.1)	198 (50.0)		6 (16.7)	23 (13.5)	
AF			0.002			0.433			0.611
No (*n*, %)	67 (38.7)	482 (52.2)		33 (45.2)	202 (51.0)		19 (52.8)	101 (59.1)	
Yes (*n*, %)	106 (61.3)	442 (47.8)		40 (54.8)	194 (49.0)		17 (47.2)	70 (40.9)	
Endocarditis			0.017			0.391			0.029
No (*n*, %)	151 (87.3)	859 (93.0)		66 (90.4)	372 (93.9)		28 (77.8)	157 (91.8)	
Yes (*n*, %)	22 (12.7)	65 (7.0)		7 (9.6)	24 (6.1)		8 (22.2)	14 (8.2)	
Cardiomyopathy			0.408			0.962			∼1.000
No (*n*, %)	162 (93.6)	882 (95.5)		70 (95.9)	376 (94.9)		34 (94.4)	164 (95.9)	
Yes (*n*, %)	11 (6.4)	42 (4.5)		3 (4.1)	20 (5.1)		2 (5.6)	7 (4.1)	
Valve			0.504			0.111			0.631
No (*n*, %)	164 (94.8)	860 (93.1)		71 (97.3)	360 (90.9)		33 (91.7)	163 (95.3)	
Yes (*n*, %)	9 (5.2)	64 (6.9)		2 (2.7)	36 (9.1)		3 (8.3)	8 (4.7)	
PVD			0.638			0.820			0.401
No (*n*, %)	150 (86.7)	816 (88.3)		64 (87.7)	340 (85.9)		33 (91.7)	165 (96.5)	
Yes (*n*, %)	23 (13.3)	108 (11.7)		9 (12.3)	56 (14.1)		3 (8.3)	6 (3.5)	
Liver			0.001			0.094			∼1.000
No (*n*, %)	154 (89.0)	885 (95.8)		63 (86.3)	368 (92.9)		35 (97.2)	169 (98.8)	
Yes (*n*, %)	19 (11.0)	39 (4.2)		10 (13.7)	28 (7.1)		1 (2.8)	2 (1.2)	
Renal			<0.001			0.939			0.061
No (*n*, %)	119 (68.8)	755 (81.7)		57 (78.1)	314 (79.3)		29 (80.6)	158 (92.4)	
Yes (*n*, %)	54 (31.2)	169 (18.3)		16 (21.9)	82 (20.7)		7 (19.4)	13 (7.6)	
Diabetes			0.007			0.966			0.921
No (*n*, %)	97 (56.1)	620 (67.1)		50 (68.5)	267 (67.4)		27 (75.0)	124 (72.5)	
Yes (*n*, %)	76 (43.9)	304 (32.9)		23 (31.5)	129 (32.6)		9 (25.0)	47 (27.5)	
Malignancy			0.004			0.062			0.268
No (*n*, %)	149 (86.1)	859 (93.0)		63 (86.3)	370 (93.4)		32 (88.9)	163 (95.3)	
Yes (*n*, %)	24 (13.9)	65 (7.0)		10 (13.7)	26 (6.6)		4 (11.1)	8 (4.7)	
Anemia			0.009			0.776			0.202
No (*n*, %)	109 (63.0)	676 (73.2)		50 (68.5)	281 (71.0)		25 (69.4)	138 (80.7)	
Yes (*n*, %)	64 (37.0)	248 (26.8)		23 (31.5)	115 (29.0)		11 (30.6)	33 (19.3)	
Thrombocytopenia			0.009			0.002			<0.001
No (*n*, %)	142 (82.1)	826 (89.4)		56 (76.7)	358 (90.4)		30 (83.3)	168 (98.2)	
Yes (*n*, %)	31 (17.9)	98 (10.6)		17 (23.3)	38 (9.6)		6 (16.7)	3 (1.8)	
Coagulopathy			<0.001			0.014			0.022
No (*n*, %)	144 (83.2)	856 (92.6)		59 (80.8)	361 (91.2)		36 (100.0)	144 (84.2)	
Yes (*n*, %)	29 (16.8)	68 (7.4)		14 (19.2)	35 (8.8)		0 (0.0)	27 (15.8)	
Delirium			0.535			0.892			∼1
No (*n*, %)	164 (94.8)	861 (93.2)		70 (95.9)	375 (94.7)		35 (97.2)	168 (98.2)	
Yes (*n*, %)	9 (5.2)	63 (6.8)		3 (4.1)	21 (5.3)		1 (2.8)	3 (1.8)	
Dementia			0.808			0.645			0.794
No (*n*, %)	162 (93.6)	882 (95.5)		70 (95.9)	371 (93.7)		36 (100.0)	167 (97.7)	
Yes (*n*, %)	11 (6.4)	42 (4.5)		3 (4.1)	25 (6.3)		0 (0.0)	4 (2.3)	
**Treatments and drugs (%)**									
Aspirin			0.178			0.867			0.562
No (*n*, %)	121 (69.9)	594 (64.3)		44 (60.3)	246 (62.1)		28 (77.8)	122 (71.3)	
Yes (*n*, %)	52 (30.1)	330 (35.7)		29 (39.7)	150 (37.9)		8 (22.2)	49 (28.7)	
Alteplase			0.749			0.767			0.775
No (*n*, %)	169 (97.7)	909 (98.4)		72 (98.6)	385 (97.2)		35 (97.2)	170 (99.4)	
Yes (*n*, %)	4 (2.3)	15 (1.6)		1 (1.4)	11 (2.8)		1 (2.8)	1 (0.6)	
Warfarin			0.195			0.487			0.289
No (*n*, %)	170 (98.3)	886 (95.9)		72 (98.6)	381 (96.2)		36 (100.0)	161 (94.2)	
Yes (*n*, %)	3 (1.7)	38 (4.1)		1 (1.4)	15 (3.8)		0 (0.0)	10 (5.8)	
Albumin			∼1			0.278			0.133
No (*n*, %)	162 (93.6)	866 (93.7)		65 (89.0)	370 (93.4)		34 (94.4)	170 (99.4)	
Yes (*n*, %)	11 (6.4)	58 (6.3)		8 (11.0)	26 (6.6)		2 (5.6)	1 (0.6)	
Epinephrine			0.965			∼1			0.001
No (*n*, %)	168 (97.1)	901 (97.5)		71 (97.3)	383 (96.7)		30 (83.3)	167 (97.7)	
Yes (*n*, %)	5 (2.9)	23 (2.5)		2 (2.7)	13 (3.3)		6 (16.7)	4 (2.3)	
Vasopressin			<0.001			0.001			0.381
No (*n*, %)	161 (93.1)	907 (98.2)		65 (89.0)	387 (97.7)		30 (83.3)	154 (90.1)	
Yes (*n*, %)	12 (6.9)	17 (1.8)		8 (11.0)	9 (2.3)		6 (16.7)	17 (9.9)	
RRT			0.001			0.643			0.618
No (*n*, %)	160 (92.5)	905 (97.9)		69 (94.5)	382 (96.5)		34 (94.4)	167 (97.7)	
Yes (*n*, %)	13 (7.5)	19 (2.1)		4 (5.5)	14 (3.5)		2 (5.6)	4 (2.3)	
MV			<0.001			<0.001			0.004
No (*n*, %)	80 (46.2)	656 (71.0)		29 (39.7)	274 (69.2)		18 (50.0)	129 (75.4)	
Yes (*n*, %)	93 (53.8)	268 (29.0)		44 (60.3)	122 (30.8)		18 (50.0)	42 (24.6)	
Scores (median [IQR])									
APSIII	65.00	39.00	<0.001	62.00	42.50	<0.001	66.50	36.00	<0.001
	[48.00, 86.00]	[29.00, 54.00]		[46.00, 83.00]	[31.00, 57.00]		[44.00, 91.75]	[26.00, 49.00]	
OASIS	40.00	31.00	<0.001	40.00	32.00	<0.001	33.00	23.00	<0.001
	[34.00, 45.00]	[25.00, 37.00]		[33.00, 46.00]	[26.00, 38.00]		[26.00, 40.25]	[18.00, 30.00]	
SOFA	6.00	3.00	<0.001	6.00	4.00	<0.001	6.00	2.00	<0.001
	[4.00, 10.00]	[2.00, 6.00]		[4.00, 7.00]	[2.00, 6.00]		[4.00, 8.00]	[1.00, 4.00]	
GCS	8.00	13.00	<0.001	9.00	12.00	0.001	8.00	13.00	<0.001
	[5.00, 13.00]	[9.00, 14.00]		[6.00, 13.00]	[8.00, 14.00]		[3.75, 12.00]	[10.00, 15.00]	

Continuous variables were expressed as the mean (standard deviation) (normal) or the median [interquartile range] (non-normal). Categorical variables were expressed as the total number(percentages). Abbreviations: AF, atrial fibrillation; APSIII, Acute Physiology and Chronic Health Evaluation III; BUN, blood urea nitrogen; CHD, coronary heart disease; CHF, congestive heart failure; COPD, chronic obstructive pulmonary disease; DBP, diastolic blood pressure; eICU-CRD, eICU Collaborative Research Database; GCS, Glasgow Coma Scale; HR, heart rate; MCH, mean corpuscular hemoglobin; MCHC, mean corpuscular hemoglobin contentration; MIMIC, Medical Information Mart for Intensive Care; MV, mechanical ventilation; OASIS, Oxford Acute Severity of Illness Score; PVD, peripheral vascular disease; RBC, red blood cell count; RDW, red cell volume distribution width; RR, respiratory rate; RRT, renal replacement therapy; SBP, systolic blood pressure; SOFA, Sequential Organ Failure Assessment; SpO_2_, peripheral oxygen saturation; WBC, white blood cell count.

### Model development and internal validation

**Figure 2:**
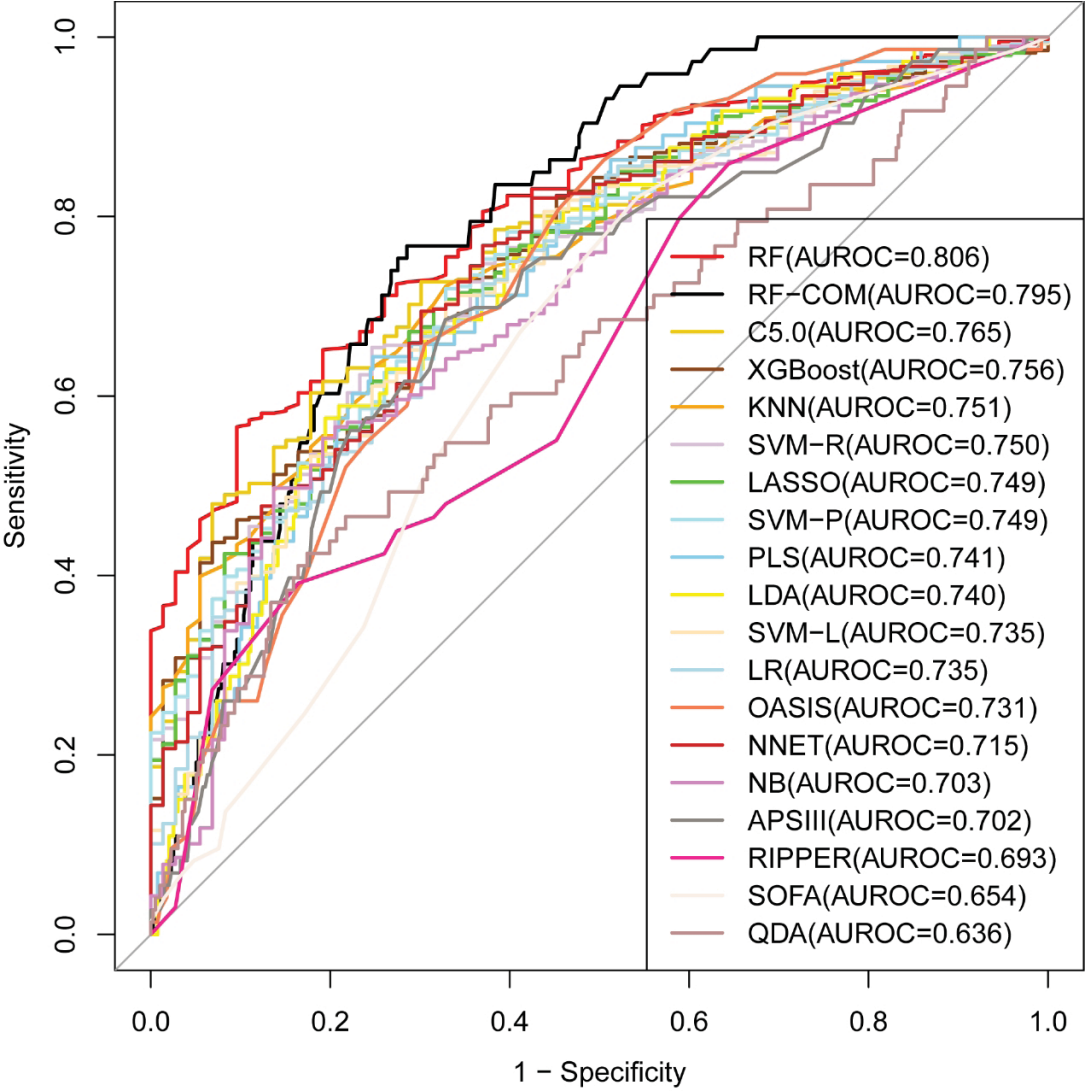
Comparison of model performance in the internal validation cohort

The training cohort was used for model development with a 3 × 10-fold cross-validation. The RF full model (i.e., all of the predictors) had the largest AUROC, AUPRC, accuracy, precision, and Cohen’s kappa value (see Supplementary Figure S1). In the internal validation phase, the RF full model still had the largest AUROC (0.806) (see Figure 2 and Table 2). Within the scoring systems, SOFA had the worst performance (AUROC = 0.654) and OASIS had an acceptable performance (AUROC = 0.731). We preferred to choose RF as the best model. The performance of RF is primarily dictated by two parameters: ntree (the number of trees) and mtry (the number of variables randomly sampled at each node). We performed extensive parameter tuning with a random combination of 30 mtry values, and manually set ntree to four values (500, 1000, 2000, 3000, and 5000). The configuration with the largest AUROC was mtry = 23 and ntree = 500.

**Table 2:** Model performance in the internal and external validation cohorts

		Internal validation								External validation							
		AUROC	BTOA	AUPRC	Accuracy	PPV	NPV	F1	Kappa	AUROC	BTOA	AUPRC	Accuracy	PPV	NPV	F1	Kappa
**Full set**	**Size**																
RF	58	**0.806**	0.183	**0.402**	0.832	**0.438**	0.876	0.350	0.260	**0.838**	0.161	0.472	0.849	0.563	0.849	0.275	0.192
C5.0	58	0.765	0.279	0.314	0.821	0.366	0.864	0.263	0.170	0.810	0.246	0.458	0.826	0.500	0.846	0.250	0.179
XGBoost	58	0.756	0.096	0.304	0.817	0.341	0.862	0.246	0.151	0.826	0.112	0.500	0.821	0.474	0.856	0.327	0.235
KNN	58	0.751	**0.635**	0.310	0.550	0.243	**0.960**	0.381	0.181	0.775	0.441	0.392	0.744	0.379	**0.928**	0.481	0.328
LASSO	28	0.749	0.454	0.318	0.706	0.290	0.911	0.395	0.232	0.821	0.268	0.485	0.821	0.487	0.899	0.507	0.398
SVM-P	58	0.749	0.460	0.327	0.721	0.307	0.915	**0.413**	0.257	0.817	0.199	0.443	0.807	0.447	0.888	0.459	0.342
SVM-R	58	0.742	0.275	0.377	0.800	0.360	0.883	0.365	0.246	0.801	0.123	0.356	0.792	0.267	0.833	0.157	0.061
PLSDA	58	0.741	0.512	0.320	0.706	0.293	0.913	0.400	0.238	0.814	0.389	0.456	0.816	0.475	0.898	0.500	0.388
LDA	58	0.737	0.623	0.319	0.706	0.293	0.913	0.400	0.238	0.817	0.395	0.483	0.811	0.465	0.902	0.506	0.391
SVM-L	58	0.735	0.426	0.323	0.719	0.294	0.905	0.389	0.230	0.808	0.204	0.463	0.840	0.541	0.906	**0.548**	**0.451**
LR	58	0.735	0.349	0.323	0.723	0.303	0.910	0.404	0.248	0.801	0.308	0.472	0.816	0.474	0.893	0.486	0.375
OASIS	10	0.731	0.393	0.301	0.653	0.263	0.918	0.380	0.200	0.754	0.202	0.369	0.783	0.395	0.884	0.430	0.297
NNET	58	0.715	0.522	0.293	0.761	0.303	0.884	0.348	0.207	0.731	0.246	0.357	0.802	0.414	0.865	0.369	0.253
NB	58	0.708	0.006	0.273	0.778	0.293	0.871	0.297	0.166	0.765	0.007	0.373	0.826	0.500	0.865	0.379	0.285
APSIII	16	0.702	0.456	0.303	0.702	0.287	0.910	0.391	0.227	0.805	0.328	0.493	0.763	0.373	0.891	0.437	0.293
RIPPER	58	0.693	0.021	0.241	0.725	0.245	0.872	0.295	0.133	0.699	0.021	0.339	0.797	0.417	0.877	0.417	0.294
SOFA	12	0.654	0.399	0.235	0.667	0.236	0.885	0.322	0.139	0.791	0.507	0.459	0.816	0.477	0.908	0.525	0.413
QDA	58	0.636	0.011	0.281	0.823	0.375	0.865	0.266	0.175	0.535	0.010	**0.797**	0.198	0.250	0.831	0.125	0.042
**RFE set**	**Size**																
RF-COM	6	0.795	0.315	0.352	0.793	0.364	0.892	0.397	0.274	0.830	0.169	0.417	0.816	0.450	0.856	0.321	0.225
RF	35	0.774	0.178	0.312	0.834	0.353	0.852	0.133	0.079	0.832	0.119	0.539	0.850	**1.000**	0.847	0.244	0.210
SVM-R	45	0.751	0.084	0.283	0.812	0.241	0.850	0.137	0.054	0.764	0.088	0.419	0.831	0.571	0.840	0.186	0.137
LR	45	0.743	0.235	0.321	0.825	0.395	0.869	0.293	0.201	0.757	0.064	0.456	0.831	0.538	0.851	0.286	0.213
SVM-L	35	0.736	0.316	0.337	0.710	0.291	0.908	0.393	0.232	0.811	0.186	0.481	0.845	0.559	0.902	0.543	0.450
PLSDA	45	0.731	0.520	0.310	0.710	0.294	0.911	0.398	0.238	0.817	0.465	0.501	0.816	0.477	0.908	0.525	0.413
QDA	10	0.720	0.023	0.256	0.785	0.281	0.864	0.263	0.137	0.796	0.201	0.449	0.836	0.545	0.870	0.414	0.325
LDA	20	0.718	0.093	0.276	0.819	0.350	0.862	0.248	0.155	0.785	0.107	0.465	0.826	0.500	0.843	0.217	0.153
XGBoost	35	0.709	0.083	0.269	0.817	0.324	0.859	0.218	**0.817**	0.815	0.088	0.472	0.831	0.533	0.854	0.314	0.236
NB	58	0.708	0.006	0.273	0.778	0.293	0.871	0.297	0.166	0.765	0.007	0.373	0.826	0.500	0.865	0.379	0.285
SVM-P	35	0.704	0.160	0.295	0.815	0.267	0.852	0.156	0.071	0.819	0.128	0.477	0.841	1.000	0.838	0.154	0.131
C5.0	45	0.701	0.097	0.269	0.821	0.333	0.858	0.208	0.122	0.773	0.102	0.473	**0.855**	0.800	0.858	0.348	0.295
RIPPER	45	0.689	0.127	0.255	0.744	0.280	0.881	0.333	0.182	0.661	0.085	0.266	0.759	0.306	0.854	0.306	0.159
KNN	20	0.637	0.218	0.277	**0.838**	0.385	0.851	0.116	0.073	0.772	0.211	0.296	0.831	0.600	0.837	0.146	0.109
NNET	10	0.572	0.615	0.190	0.567	0.165	0.851	0.240	0.018	0.687	**0.580**	0.306	0.289	0.916	0.826	0.413	0.219

Models are ordered according to their area under the receiver operating characteristic curve (AUROC) in the internal validation set. The bold values indicate the largest value in the internal or external validation.

Abbreviations: APSIII, acute physiology and chronic health evaluation III; AUPRC, area under the precision-recall curve; AUROC, area under the receiver operating characteristic curve; BTOA, best threshold of AUROC; COM, compact; KNN, *k*-nearest neighbor; LASSO, least absolute shrinkage and selection operator; LDA, linear discriminant analysis; LR, logistic regression; NB, naive bayes; NNET, neural network; NPV, negative predictive value; OASIS, oxford acute severity of illness score; PLSDA, partial least squares discriminant analysis; PPV, positive predictive value; QDA, quadratic discriminant analysis; RF, random forest; RFE, recursive feature elimination; RIPPER, repeated incremental pruning to produce error reduction; SOFA, sequential organ failure assessment; SVM, support vector machine; SVM-L, SVM with linear kernel; SVM-P, SVM with a polynomial kernel; SVM-R, SVM with radial basis function kernel; XGBoost, extreme gradient boosting machine.

### Feature selection and the subset model

**Figure 3:**
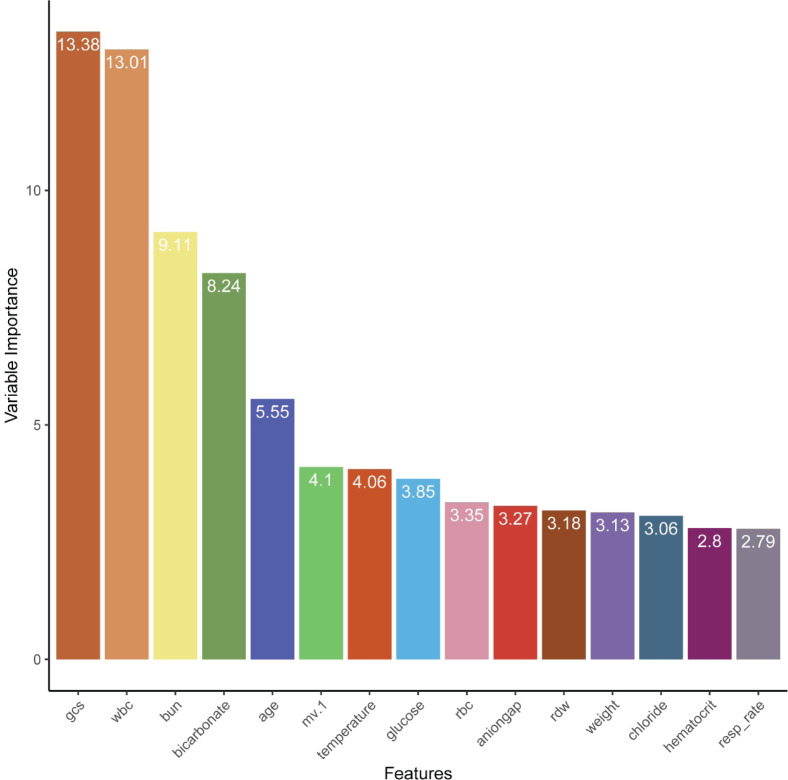
The top 15 key features identified by recursive feature elimination coupled with random forest

Supplementary Figure S2 showed results for each ML model generated by the RFE process in the training set. Of the evaluated models, the RF model (labeled as RF-RFE) performed best (AUROC = 0.859), and the screened feature subset counted 35 features. Figure 3 showed the top 15 key features. Despite some minor inconsistencies, the internal validation results showed trends that were comparable to those associated with the training results. The RF-RFE model retained largest AUROC (=0.774) among all RFE models (see Table 2). Generally, an AUROC of 0.9–1.0 is considered outstanding, 0.8–0.9 is considered good, 0.7–0.8 is considered fair, and 0.6–0.7 is considered poor [36]. Therefore, based on the clinical availability, we selected the minimum feature subset of AUROC > 0.840 to develop an RF compact model (labeled as RF-COM) with the top six features (GCS, WBC, BUN, bicarbonate, age, and MV). In the internal validation phase, the AUROC of the RF-COM model was 0.795, only second to the RF full model (AUROC = 0.806) (see Figure 2 and Table 2).

### External validation

**Figure 4:**
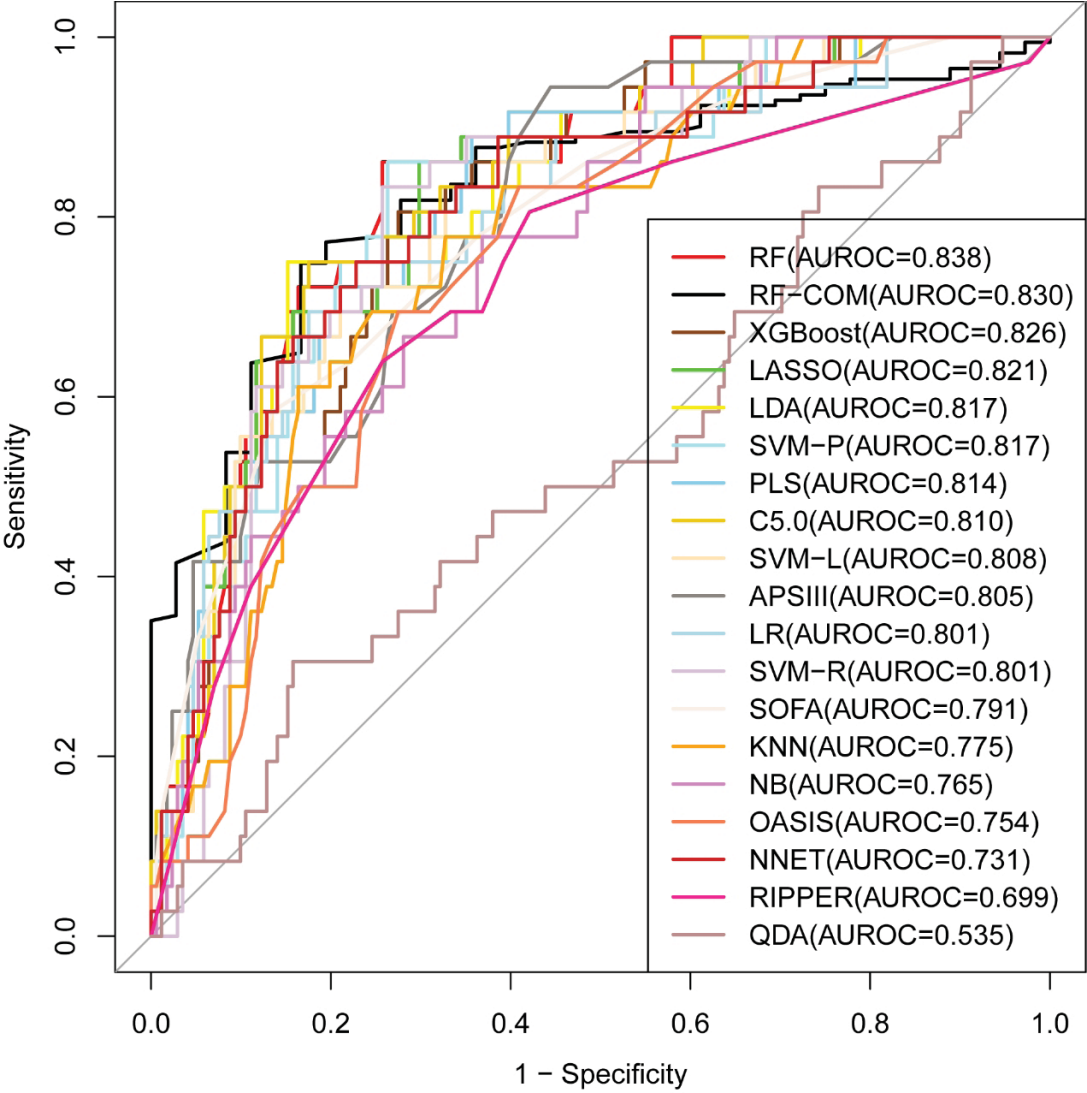
Comparison of model performance in the external validation cohort

The external validation results of full models and RFE models are presented in Figure 4 and Table 2. Almost all models had larger AUROC than the internal validation. The RF full model still performed the best (AUROC = 0.838), followed by the RF-RFE model (AUROC = 0.832) and RF-COM model (AUROC = 0.830). Among the scoring systems, OASIS performed the worst (AUROC = 0.754), with smaller AUROC than APSIII (AUROC = 0.805) and SOFA (AUROC = 0.791).

### Explanation of the model at the individual level

**Figure 5:**
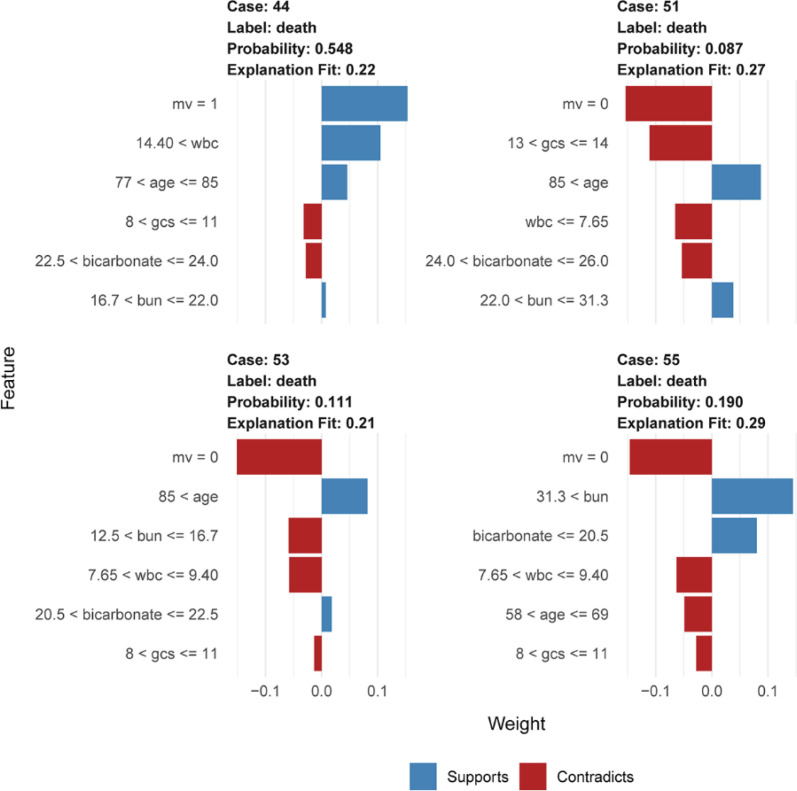
LIME plots of four representative instances Results of LIME with RF-COM model applied to one positive (case 44) and three negative instances (case 51, case53, and case 55). The blue indicates a condition that supports death, and the red indicates a condition that does not support death. mv = 1 represents the use of mechanical ventilation, mv = 0 represents no use of mechanical ventilation

LIME plots were used to illustrate the impact of key features on individual patients. We presented four cases whose mortality had been correctly predicted using the RF-COM model in the internal validation cohort (see Figure 5). Case 44 from the ‘true positive’ group was correctly predicted for death, and the other three cases from the ‘true negative’ group were correctly predicted for survival. Taking case 44 as an example, the death probability was 0.548 owing to the influence of the support conditions, including the required MV, WBC > 14.4 × 10^9^/L (= 19.24 × 10^9^/L), and age > 77 years (= 83 years).

## Discussion

By developing ML models, the present study identified the mortality predictors for ES patients in ICU. We combined ML with feature selection methods to determine the best ML model and the key predictors for predicting mortality of the critically ill patients with ES. Our result showed that the RF model exhibited the best prediction accuracy, stability, and generalization. RF is a powerful ensemble learning algorithm. It has several advantages, including high accuracy, robustness to overfitting, estimation of important features, and handling unbalanced and missing data [37]. Compared with hundreds of other ML algorithms applied to many datasets, RF has emerged as the best performer overall [38]. RF has also shown a high degree of accuracy in mortality prediction for a plethora of diseases in ICU, such as acute kidney injury [39], acute respiratory distress syndrome [40], and cerebral hemorrhage [41].

The RF full model comprises 58 features and the RF-RFE model comprises 35 features. Evaluating more than 30 variables at once is time-consuming and may not be feasible in conventional clinical practice. As reported in a previous study, 37.3 min on average were required to perform a complete APACHE III assessment with several tens of variables [42]. There is no doubt that this is a heavy clinical workload for a clinician. A model with lesser features could be more clinically applicable and less expensive from a practical standpoint. Therefore, we constructed an RF-COM model with the top six features and discovered that the RF-COM model has approximately the same predictive power as the RF full model.

We then compared the features in the RF-COM model with those in the three scoring systems (see Supplementary Table S3). There are 16 features in APSIII, 12 in SOFA, and 10 in OASIS. GCS, mean arterial pressure (MAP), and urine output (24 h) are common recognition features among the scoring systems. Of the six features of RF-COM model, three features were included in APSIII (GCS, WBC, and BUN), three features in OASIS (GCS, age, and MV), and two features in SOFA (GCS and MV). GCS is not only the feature commonly identified by RF-COM model and scoring systems but also the most important feature identified by RF-COM model. GCS is used as a measure of consciousness and has the advantages of speed and ease of evaluation. A large number of studies have found that GCS is a strong predictor of hospital mortality and poor neurological outcome [26,27,43], which might be the reason that most of the scoring systems use GCS as the neurological assessment tool rather than other neurological scales, such as the National Institute of Health Stroke Scale (NIHSS).

When we tested the predictive performance of GCS as a univariate predictor, we discovered that GCS has comparable performance with the three scoring systems. The AUROC is 0.626 in the internal validation cohort, slightly lower than SOFA (= 0.654), and the AUROC is 0.757 in the external validation cohort, slightly higher than OASIS (= 0.754). Interestingly, similar findings have been found in some other studies. Bhagwanjee et al*.* evaluated APACHE II and GCS for mortality prediction in 105 cases of severe eclampsia and concluded that the prediction performance were very similar with both methods [14]. Cho et al*.* used APACHE II, APACHE III, and GCS to predict the mortality of 200 patients with acute craniocerebral injury and found that GCS had almost the same predictive ability as the other two scoring systems [13]. Raj et al*.* found that a simple model based on GCS and age was comparable to APACHE II, SAPS II, and SOFA in predicting 6-month mortality in patients with traumatic brain injury [15].

We then investigated the five other features and found that they had all been reported as the predictors of mortality for stroke and other diseases. For example, several studies have shown that acute stroke patients requiring MV have high in-hospital mortality and survival patients remain deeply disabled [44,45]. BUN is usually considered a less specific marker of renal function. It is independently associated with mortality in many diseases [46,47]. Bicarbonate has been reported to be associated with mortality from various diseases, which can be attributed to numerous factors in most cases [48–50]. An increasing number of studies have focused on discovering the association between high WBC and the poor outcome of IS [47,51]. Inflammation and stress responses caused by IS have been proposed as plausible explanations for the association [45]. Age was found to be a strong predictor of death after IS [52,53].

Subsequently, we compared the prediction power of the top six features by univariable analysis. Surprisingly, the AUROC of GCS was not the largest in either internal or external validation phrases (see Supplementary Figure S3). This result reminds us that (i) the predictive power of a feature is different in univariate and multivariate analyses, (ii) the predictive power of a feature is limited, and the combination of multiple features using an appropriate algorithm is better than that of any single feature. In the present study, although the AUROC of GCS is not dissimilar to that of the three scoring systems, it is significantly lower than the RF-COM model with six features. Therefore, the most critical step is finding the appropriate algorithm and combination of appropriate feature subsets. According to the ‘no free lunch’ theorem, no algorithm can always perform better than others [54]. Researchers need to put more effort into systematically exploring a wide variety of algorithms and testing their performance of different subsets of features. The present study attempted to realize such an approach. By combining multiple features, the RF model improved the predictive power of hospital mortality for ES patients significantly better than a single feature and the scoring systems.

Furthermore, there are several other advantages of the study. First, the MIMIC-IV and eICU-CRD databases are publicly available, high-quality, and large-scale. To the best of our knowledge, a total of 1763 (1556 + 207) ES samples is a considerable sample size in the population-based study of ES. Second, this is the first study on the prediction of ES mortality using ML models. We compared a representative set of 15 ML models and three scoring systems and optimized their performances using different preprocessing methods for different models, thus allowing a more objective selection of the best model. Third, the top six features identified by RFE are easily obtained and assessed in clinical practice. We modeled them and performed rigorous internal and external validation. The good predictive performance of the RF-COM model confirmed the six features’ reliability as key mortality predictors for ES patients. Fourth, to provide clinicians with reliable insight into the key predictors, we used LIME to show each feature’s contribution to individual predictions.

At the same time, we must acknowledge that our study has some limitations. First, although the two databases are of high quality, they still have a substantial amount of missing data. Thus we had to drop many variables, which may lead to losing some important predictors for predicting ES mortality. Second, the two databases are non-neurospecialty ICU databases; therefore, some variables that may be useful for assessing the condition of ES patients are not available, such as NIHSS, infarct volume, and infarct location. Third, internal validation typically outperforms external validation. However, in this study, the AUROC of external validation exceeded that of internal validation in almost all ML models. In our view, the main reason may be that the AUROC of top two features (BUN = 0.771, GCS = 0.757) in the external validation set was significantly larger than the top two features (WBC = 0.660, MV = 0.647)) in the internal validation set (*P*<0.05). And the larger AUROC of the top two features may result from the more significant statistical differences between the death and survival groups in the external validation set than in the internal validation set (see Table 1). Fourth, the results were based on a standard ML prediction modeling in the present study. We did not intensively investigate the detailed mechanism of the six features associated with ES mortality. Future studies are needed to explore the underlying mechanism. Finally, although AUROC is currently regarded as the best and most commonly used evaluation metrics for binary classification models, it is only an index of comprehensive evaluation of sensitivity and specificity, and cannot reflect the degree of clinical harm caused by missed diagnosis (false negative) or misdiagnosis (false positive). Future clinical confirmatory research may need to select appropriate cutoff points based on individual characteristics of specific patients, the clinical impact of predicted results, economic cost and other factors. For example, in order to reduce the waste and disarray of medical resources caused by misdiagnosis, a cutoff point associated with higher specificity may represent a more effective choice. In order to mitigate delayed diagnosis caused by initially missed diagnosis, a cutoff point associated with higher sensitivity may represent a more appropriate choice. Only in specific patients and under specific conditions can the applicability of the predictive model be engaged to its full extent.

## Conclusion

The RF model outperformed other ML models and scoring systems in terms of accuracy, stability, and generalization. GCS, WBC, BUN, bicarbonate, age, and MV are the key mortality predictors for critically ill ES patients. The findings of this study provide clinicians with insights; however, further validation in prospective cohorts is required before they can be considered clinically acceptable.

## Supplementary Material

Supplementary Material Figure S1-S3 and Tables S1-S3

## Data Availability

Publicly available datasets were analyzed in this study. These data can be found here: https://mimic.mit.edu/iv/; https://eicu-crd.mit.edu/.

## Abbreviations

APACHE III: Acute Physiology and Chronic Health Evaluation III
APSIII: Acute Physiology Score III
AUPRC: area under the precision-recall curve
AUROC: area under the receiver operating characteristic curve
eICU-CRD: eICU Collaborative Research Database
ES: embolic stroke
GCS: Glasgow Coma Scale
ICU: intensive care units
IQR: interquartile range
IS: ischemic stroke
KNN: *k*-nearest neighbor
LASSO: least absolute shrinkage and selection operator
LDA: linear discriminant analysis
LIME: local interpretable model-agnostic explanations
LR: logistic regression
MIMIC: Medical Information Mart for Intensive Care
ML: machine learning
MV: mechanical ventilation
NB: naive bayes
NIHSS: National Institute of Health Stroke Scale
NNET: neural network
NPV: negative predictive value
OASIS: Oxford Acute Severity of Illness Score
PLSDA: partial least squares discriminant analysis
PPV: positive predictive value
QDA: (quadratic discriminant analysis)
RF: random forest
RFE: recursive feature elimination
RIPPER: repeated incremental pruning to produce error reduction
SD: standard deviation
SMOTE: synthetic minority oversampling technology
SOFA: Sequential Organ Failure Assessment
SVM: support vector machine
TRIPOD: Transparent Reporting of a multivariable prediction model for Individual Prognosis Or Diagnosis
XGBoost: extreme gradient boosting machine
