# Supplementary material for: Identification of key predictors of hospital mortality in critically ill patients with embolic stroke using machine learning
Source: Biosci Rep. 2022 Sep 14;42(9):BSR20220995. doi: 10.1042/BSR20220995 (PMC9484010; doi:10.1042/BSR20220995)
Supplement: Supplementary Material Figure S1-S3 and Tables S1-S3 [file BSR-2022-0995_supp.pdf]

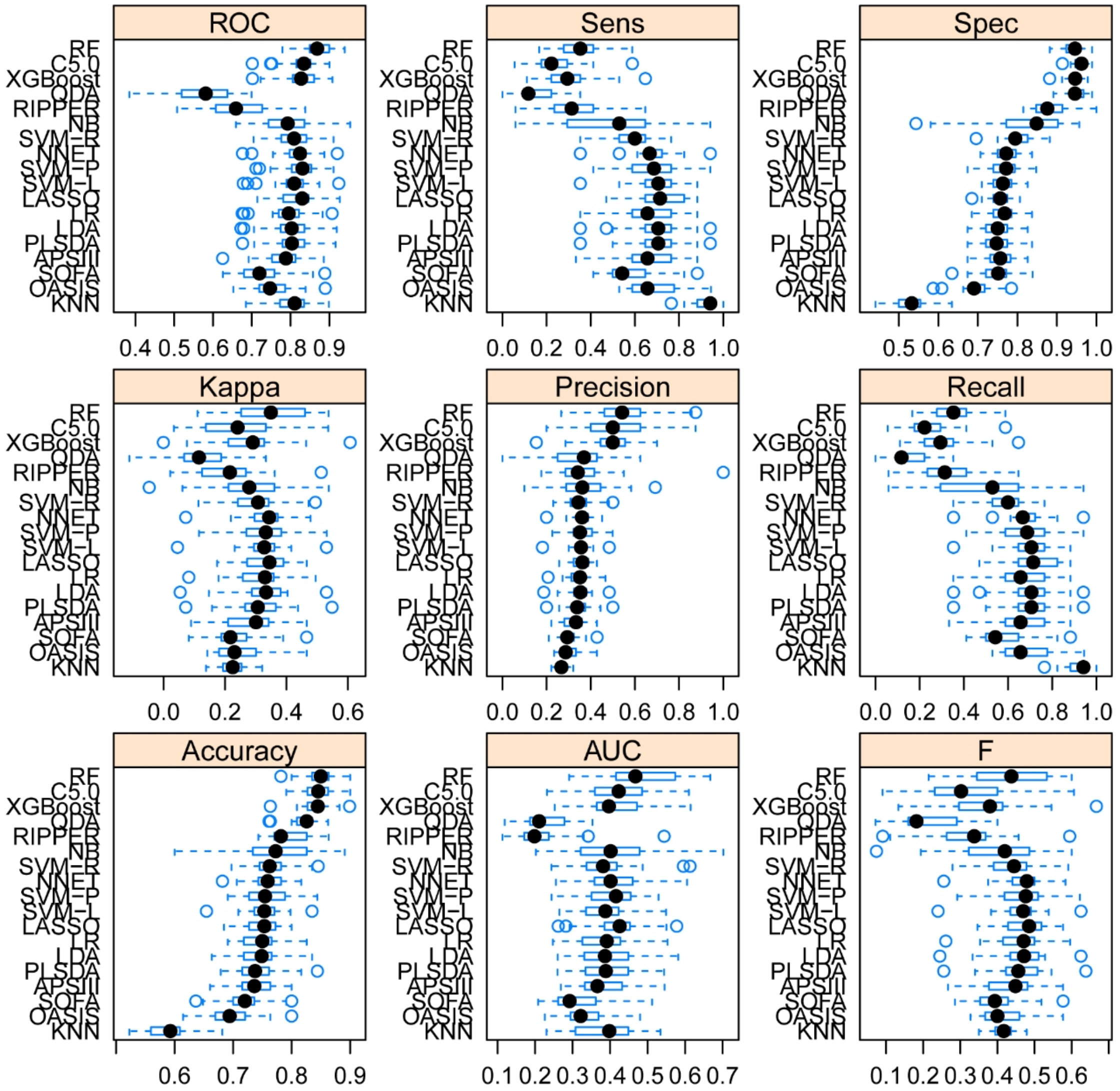

Supplementary Figure 1. Comparison of AUROC among the models in the training cohort.

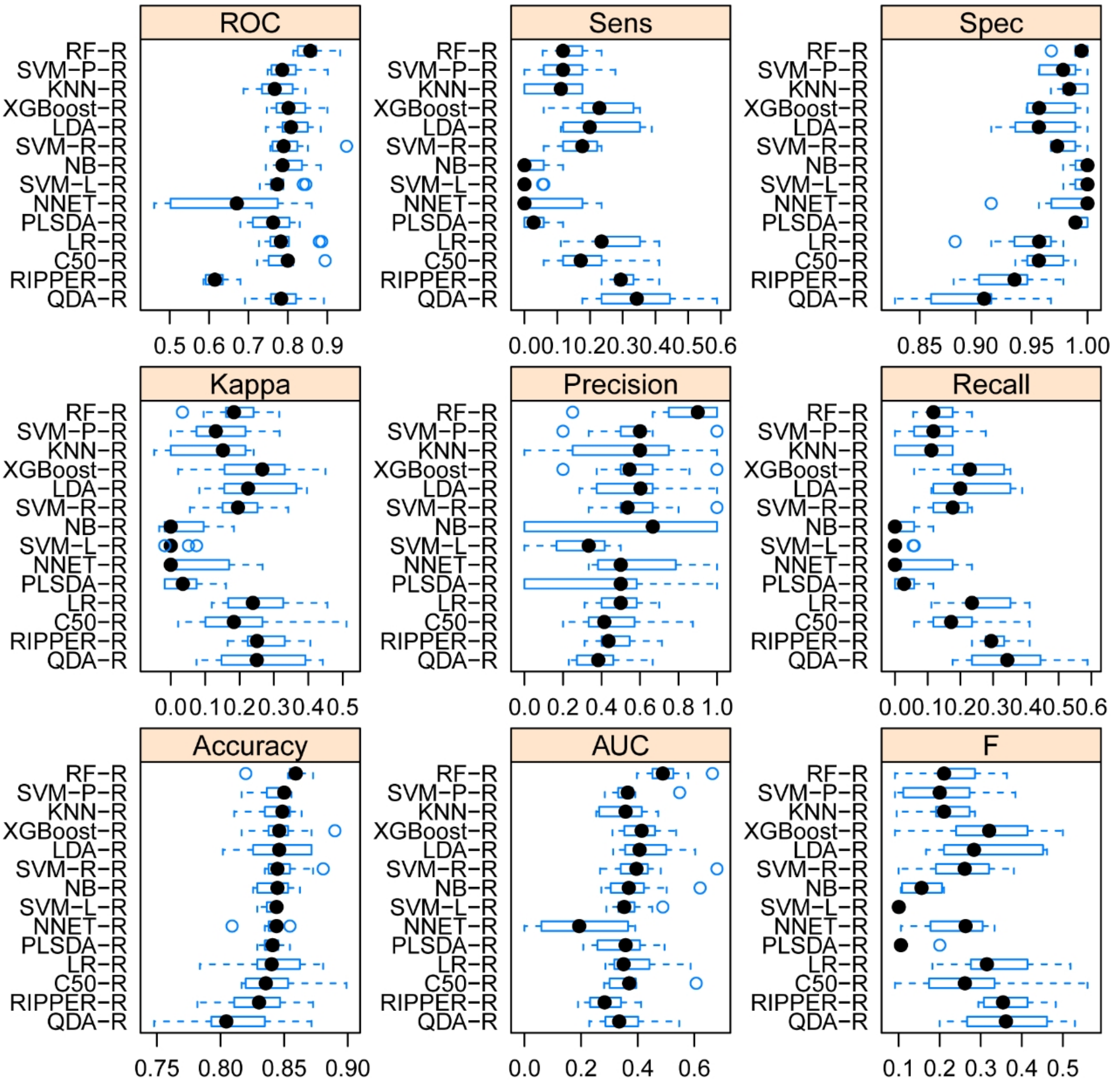

Supplementary Material Figure 2. Comparison of AUROC among the 14 machine learning models for recursive feature selection in the training cohort.

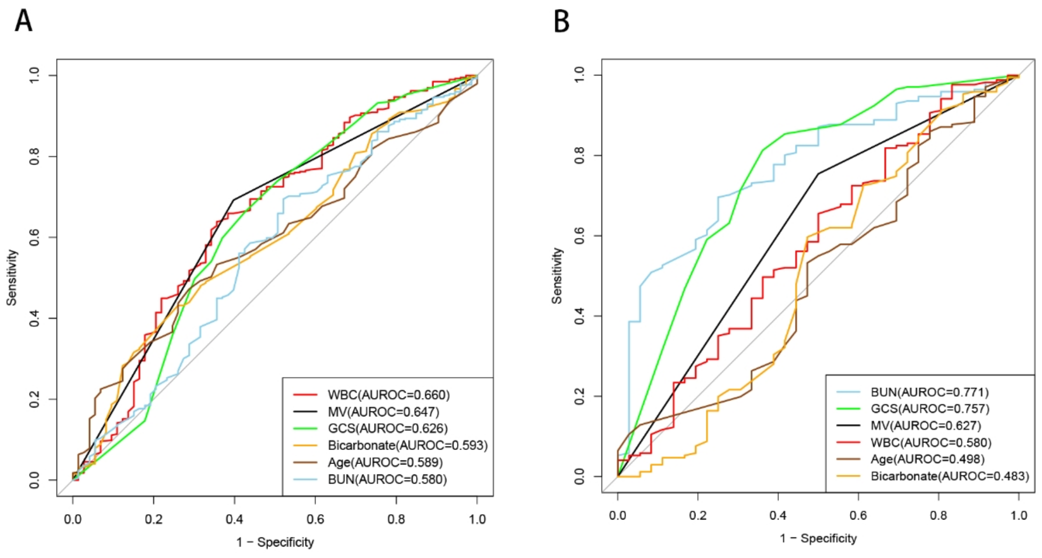

Supplementary Material Figure 3. Comparison of predictive ability among the top six features.

(A) AUROC in the internal validation cohort. (B) AUROC in the external validation cohort

Supplementary Material Table 1. Search ICD codes or terms of embolic stroke in the MIMIC-IV and eICU-CRD databases

Database	Search codes or terms
MIMIC-IV	ICD-9:
	43411:
	ICD-10:
	I6310, I6311, I63111, I63112, I63113, I63119, I6312, I6313, I63131, I63132, I63133, I63139, I6319, I634, I6340, I6341, I63411, I63412, I63413, I63419, I6342, I63421, I63422, I63423, I63429, I6343, I63431, I63432, I63433, I63439, I6344, I63441, I63442, I63443, I63449, I6349
eICU-CRD	ischemic stroke|cardioembolic
	ischemic stroke|atheroembolic

ICD, International Classification of Diseases; MIMIC, Medical Information Mart for Intensive Care; eICU-CRD, eICU Collaborative Research Database; ICD, International classification of diseases.

Supplementary Material Table 2. Predictors extracted from MIMIC-IV and eICU-CRD databases

Variable type	Variable
Demographic characteristics	Age, Weight, Gender, Ethnicity
Vital signs	SBP, DBP, HR, RR, Temperature, SpO_2
Laboratory tests	WBC, RBC, MCH, MCHC, RDW, Hematocrit, Hemoglobin, Platelets, Aniongap, Bicarbonate, BUN, Creatinine, Glucose, Chloride, Sodium, Potassium, Calcium
Comorbidities	COPD, CHD, CHF, Hypertension, Hyperlipidemia, AF, Endocarditis, Cardiomyopathy, Valve Disease, PVD, Liver Disease, Renal Disease, Diabetes, Malignancy, Anemia, Thrombocytopenia, Coagulopathy, Delirium, Dementia
Drugs and treatments	Aspirin, Alteplase, Warfarin, Albumin, Epinephrine, Vasopressin, RRT, MV
Scoring systems	GCS, APSIII, OASIS, SOFA

MIMIC, Medical Information Mart for Intensive Care; eICU-CRD, eICU Collaborative Research Database; SBP, systolic blood pressure; DBP, diastolic blood pressure; HR, heart rate; RR, respiratory rate; SpO_2, peripheral oxygen saturation; WBC, white blood cell count; RBC, red blood cell count; MCH, mean corpuscular hemoglobin; MCHC, mean corpuscular hemoglobin contentration; RDW, red cell volume distribution width; BUN, blood urea nitrogen; COPD, chronic obstructive pulmonary disease; CHD, coronary heart disease; CHF, congestive heart failure; AF, atrial fibrillation; PVD, peripheral vascular disease; RRT, renal replacement therapy; MV, mechanical ventilation; GCS, Glasgow Coma Scale; APSIII, Acute Physiology and Chronic Health Evaluation III; OASIS, Oxford acute severity of illness score; SOFA, Sequential Organ Failure Assessment.

Supplementary Material Table 3. Variable comparisons between the RF-COM model and the scoring systems

APSIII	SOFA	OASIS	RF-COM
GCS	GCS	GCS	GCS
WBC	MV	MV	WBC
BUN	Platelets	Age	BUN
Respiratory rate	Bilirubin	Heart rate	Bicarbonate
PaO_2/ $P_{A\text{-}a}O_2$	MAP	MAP	Age
Hematocrit	Dopamine	Respiratory rate	MV
Heart rate	Dobutamine	Temperature	
Creatinine	Adrenaline	Urine output	
Urine output	Norepinephrine	Pre-ICU LOS	
MAP	Creatinine	Elective surgery	
Sodium	Urine output		
Albumin	PaO2/FiO2		
Bilirubin			
Glucose			
pH			
Temperature			

RF-COM model, random forest compact model; APSIII, Acute Physiology Score III; SOFA, Sequential Organ Failure Assessment; OASIS, Oxford Acute Severity of Illness Score; MAP, mean arteriai pressure; PaO_2, Partial pressure of arterial oxygen; $P_{A\text{-}a}O_2$, alveolar-arterial PO_2 difference; WBC, white blood cell count; BUN, blood urea nitrogen; GCS, Glasgow Coma Scale; LOS, length of stay; MV, mechanical ventilation.
